# Early pubertal timing is associated with lower sperm concentration in college students

**DOI:** 10.18632/oncotarget.24415

**Published:** 2018-02-06

**Authors:** Xiaogang Wang, Peng Zou, Min Mo, Huan Yang, Qing Chen, Niya Zhou, Lei Sun, Hongqiang Chen, Lin Ao, Zhihong Cui, Jia Cao

**Affiliations:** ^1^ Key Lab of Medical Protection for Electromagnetic Radiation, Ministry of Education of China, Institute of Toxicology, College of Preventive Medicine, Third Military Medical University, Chongqing, China

**Keywords:** puberty, semen quality, reproductive hormones, andrology, epidemiology, Pathology

## Abstract

To study the associations between pubertal timing and semen quality and reproductive hormones, 680 volunteers were recruited from universities in Chongqing, China. Pubertal timing was obtained using a questionnaire. The main measurements were five routine semen parameters and six reproductive hormones. After adjusting for potential confounders, we found that early pubertal timing was associated with lower sperm concentration. An one-year increase in age of peak height velocity was associated with a 4.7% (95% confidence interval [CI] = 1.0 to 8.6) increase in sperm concentration. An one-year increase in age of first spermatorrhea was associated with a 6.4% increase in sperm concentration and a 2.9% decrease in semen volume (95% CI = 1.7 to 11.3, −5.5 to −0.3; respectively). Regarding reproductive hormones, an one-year increase in age of height spurt and peak height velocity was associated with a 6.5% and a 6.7% decrease in estrogen (95% CI = −9.8 to −3.0, −10.4 to −2.8; respectively). While an one-year increase in age of height spurt was associated with higher follicle-stimulating hormone (% change = 2.6, 95% CI = 0.2 to 4.7). This was the first report that has suggested that early pubertal timing is associated with lower sperm concentration. However, further study is still needed to validate this association and fully elucidate the mechanism behind it.

## INTRODUCTION

Puberty is a critical period as one moves from childhood to reproductive maturity [1]. During this period, hypothalamic-pituitary-gonadal (HPG) axis is triggered, culminating in adult hormonal profiles and physical changes, including growth spurts, testis development, and first spermatorrhea in boys [2]. The onset ages of these pubertal events can vary from 9 to 14 years, and the timing of each event is highly variable among boys [3]. Some studies have shown that pubertal timing is associated with later adverse health outcomes, such as diabetes [3], cardiovascular disease [4, 5], hormone-related cancer [6–8], testicular cancer [9] and other conditions. Pubertal timing has been consistently reported to have become earlier and earlier in recent decades [10, 11]. Thus, a better understanding of the association between pubertal timing and health outcome is of great significance.

A great many studies have reported that human semen quality has declined over the past decades [12, 13]. Researchers are now making an earnest effort to explore the risk factors for male reproductive health. As well known, puberty is an important process for reproductive development and reproductive-related-hormone establishment. Therefore, pubertal timing is very likely to be associated with male reproductive health. Semen quality and reproductive hormones are also well-known predictors for male reproductive health. Although the power of these parameters to evaluate a man's fertility is still limited, they have been found to be very helpful in diagnosing male infertility [14]. Routine semen parameters are widely used in research and clinical application [15]. Sex hormones, such as estrogen (E_2_) and follicle-stimulating hormone (FSH), were crucial for the development and maintenance of fertility [16, 17].

The Male Reproductive Health in Chongqing College Students (MARHCS) study was established to investigate the effects of environmental and socio-psycho-behavioral factors on male reproductive health [18–21]. Based on a population of 680 young male college students in Chongqing China, we explored that whether pubertal timing was associated with semen quality and reproductive hormones.

## RESULTS

### The characteristics of subjects

The demographic characteristics of the study participants are presented in Table 1. The volunteers were all young adults with a median (25th to 75th percentile) age of 20 years (20 to 21). Their abstinence time (median was 4 days) was kept between 2 to 7 days. In terms of personal lifestyle factors, most had never smoked before (75.8%). About half drank alcohol (47.6%). About 35.1% didn't drink Coke. Tea and coffee were not popular. The parameters on semen quality and reproductive hormones were presented as median (25th to 75th percentile). Parameters on semen quality were dichotomized according to the WHO Guideline 2010 [15]. The normal percentages of semen volume, sperm concentration, total sperm number, progressive motility, total motility, and morphological normal spermatozoa were 96.5%, 94.7%, 95.0%, 92.6%, 99.9%, 95.3%, respectively.

**Table 1:** Characteristics of the subjects according to the onset age of peak height velocity

Characteristicsa	Onset age of peak height velocity				
	Total	≤25th percentile	25th–75th percentile	>75th percentile	*P* value^b^
No. of subjects	680	193	291	127	
Demographic characteristics					
Age, years	20 (20, 21)	20 (20, 21)	20 (20, 21)	20 (20, 21)	0.023
Abstinence period, days	4 (3, 6)	4 (3, 6)	4 (3, 6)	4 (3, 5)	0.534
Body mass index, kg/m^2^					0.014
<18.5	68 (10.0%)	15 (7.8%)	29 (10.0%)	13 (10.3%)	
18.5–23.9	522 (76.9%)	137 (71.0%)	232 (79.7%)	102 (81.0%)	
24-27.9	73 (10.8%)	33 (17.1%)	26 (8.9%)	8 (6.3%)	
≥28	16 (2.4%)	8 (4.1%)	4 (1.4%)	3 (2.4%)	
Tobacco smoking					0.323
Never	515 (75.8%)	145 (75.5%)	219 (75.3%)	97 (76.4%)	
Ever	29 (4.3%)	6 (3.1%)	10 (3.4%)	9 (7.1%)	
Current	135 (19.9%)	41 (21.4%)	62 (21.3%)	21 (16.5%)	
Alcohol consumption					0.540
Never	345 (50.9%)	98 (51%)	145 (49.8%)	64 (50.4%)	
Ever	10 (1.5%)	2 (1.0%)	6 (2.1%)	0 (0.0%)	
Current	323 (47.6%)	92 (47.9%)	140 (48.1%)	63 (49.6%)	
Tea intake					0.128
Never	438 (64.5%)	111 (57.8%)	197 (67.7%)	87 (68.5%)	
Ever	112 (16.5%)	39 (20.3%)	38 (13.1%)	19 (15.0%)	
Current	129 (19.0%)	42 (21.9%)	56 (19.2%)	21 (16.5%)	
Cola intake, bottles/week					0.133
0	238 (35.1%)	54 (28.1%)	109 (37.5%)	51 (40.2%)	
<3	343 (50.5%)	109 (56.8%)	144 (49.5%)	53 (41.7%)	
3–6	84 (12.4%)	25 (13.0%)	32 (11.0%)	21 (16.5%)	
>6	14 (2.1%)	4 (2.1%)	6 (2.1%)	2 (1.6%)	
Coffee intake, cups/week					0.642
0	511 (75.3%)	140 (72.9%)	215 (73.9%)	104 (81.9%)	
<3	137 (20.2%)	41 (21.4%)	63 (21.6%)	19 (15.0%)	
3–6	18 (2.7%)	6 (3.1%)	7 (2.4%)	2 (1.6%)	
>6	13 (1.9%)	5 (2.6%)	6 (2.1%)	2 (1.6%)	
Testicular volume, ml	17 (15, 20)	17 (15, 20)	17 (15, 20)	16.8 (15, 20)	0.734
Semen parameters					
Sperm volume, ml	3.4 (2.5, 4.5)	3.4 (2.3, 4.7)	3.5 (2.6, 4.4)	3.3 (2.5, 4.6)	0.750
Sperm concentration, millions/ml	54.9 (32.6, 84.8)	51.1 (27.3, 81.3)	56.3 (35.7, 85.0)	55.5 (35.7, 88.2)	0.102
Total sperm number, millions	187.0 (102.2, 300.5)	163.1 (91.5, 287.4)	198.3 (105.1, 318.9)	184.5 (130.3, 302.3)	0.123
Progressive motility,%	56.2 (43.6, 68.6)	58.5 (45.6, 68.9)	54.2 (42.3, 69)	55.2 (43.4, 68.8)	0.330
Total motility, %	89.1 (79.9, 94.9)	89.6 (80.9, 95.4)	88.3 (79.3, 94.8)	89.5 (80.1, 94.5)	0.478
Morphological normal spermatozoa, %	8.3 (6.4, 10.3)	8.0 (6.0, 9.9)	8.5 (6.5, 10.5)	8.4 (6.5, 9.9)	0.179
Reproductive hormones					
Estradiol, pg/ml	19 (10, 27)	20 (12, 30)	18 (10, 27)	16 (9, 26)	0.056
Follicle-stimulating hormone, mIU/ml	3.5 (2.6, 4.9)	3.4 (2.6, 4.7)	3.5 (2.6, 4.6)	3.6 (2.9, 5.5)	0.108
Luteinizing hormone, mIU/ml	4.1 (3.1, 5.1)	4.0 (3.0, 5.2)	3.9 (3.1, 5.2)	4.1 (3.1, 4.9)	0.999
Progesterone, ng/ml	10.0 (8.1, 13.5)	9.9 (8.4, 14.0)	10.3 (8.1, 13.2)	9.8 (7.7, 13.3)	0.540
Testosterone, ng/ml	0.5 (0.3, 0.8)	0.5 (0.3, 0.8)	0.5 (0.3, 0.8)	0.5 (0.3, 0.8)	0.973
Prolactin, ng/ml	4.3 (3.6, 5.1)	4.3 (3.5, 5.1)	4.3 (3.6, 5.1)	4.3 (3.5, 5.2)	0.984

^a^Represented as “median (25th, 75th percentile)” or “number (percentage)”.

^b^Kruskal-Wallis tests for continuous variables and Chi-square tests for categorical variables.

### Descriptive statistics of onset ages of pubertal events

A total of 680 volunteers completed the questionnaire on puberty (Table 2). However, not every question listed on the questionnaire was answered by the participants. The most frequently answered question was age of height spurt (*N* = 627, 92.2%) (Table 2), and least frequently answered question was age of first spermatorrhea (*N* = 508, 74.7%). All ages regarding puberty events had a high internal consistency. Overall, the raw Cronbach's alpha coefficient was 0.886, and the practical cutoff was 0.7. The mean ages of pubertal events in this cohort ranged from 13.0 to 15.4 years. The order of pubertal events in men was: height spurt, body hair growth, first spermatorrhea, peak height velocity, voice deepening, skin changes, and facial hair growth. In addition, onset ages of these pubertal events were significantly correlated with each other (Supplementary Table 2). The correlation coefficients ranged from 0.356 to 0.738 (*P* < 0.001).

**Table 2:** Descriptive statistics of onset ages of pubertal events

Pubertal events (y)	*N*	Mean	Standard Deviation	Median	Percentile 25	Percentile 75
Height spurt	627	13.0	2	13	12	14
Body hair growth	560	13.5	1	13	13	14
First spermatorrhea	508	13.8	1	14	13	15
Peak height velocity	626	14.2	2	14	13	15
Voice deepening	565	14.4	2	14	13	15
Skin changes	580	14.7	2	14	13	16
Facial hair growth	586	15.4	2	15	14	17

### Associations between pubertal timing and semen quality and reproductive hormones

We used multivariate regression to assess the associations between the onset ages of pubertal events, semen quality (except progressive motility and total motility) and reproductive hormones (Table 3). After adjusting for potential confounders, an one-year increase in age of peak height velocity was associated with a 4.7% increase in sperm concentration (95% Confidence Interval (CI) = 1.0 to 8.6) and a 2.1% increase in morphologically normal spermatozoa (95% CI = 0.3 to 4.0). An one-year increase in age of first spermatorrhea was also associated with a 6.4% (95% CI = 1.7 to 11.3) increase in sperm concentration and a 2.9% decrease in semen volume (95% CI = **−**5.5 to **−**0.3). There was no such association between total sperm count, progressive motility, total motility and the onset ages of pubertal events.

**Table 3:** Associations between onset ages of pubertal events, semen quality and reproductive hormones

Characteristics^a,b^	Height spurt, % Change (95%CI)	Peak height velocity, % Change (95%CI)	Body hair growth, % Change (95%CI)	Skin changes, % Change (95%CI)	Voice deepening, % Change (95%CI)	Facial hair growth, % Change (95%CI)	First spermatorrhea, % Change (95%CI)
Semem quality							
Sperm concentration, millions/ml	3.3 (–0.4, 7.1)	4.7 (1.0, 8.6)^*^	0.9 (–3.5, 5.6)	2.3 (–1.3, 6.1)	2.3 (–1.7, 6.6)	1.9 (–1.8, 5.6)	6.4 (1.7, 11.3)^**^
Semen volume, ml	–0.2 (–2.5, 2.0)	0.0 (–2.2, 2.3)	0.0 (–2.7, 2.7)	1.2 (–1.1, 3.5)	0.0 (–2.2, 2.3)	–0.2 (–2.0, 1.6)	–2.9 (–5.5, –0.3)^*^
Total sperm count, millions	2.3 (–1.7, 6.6)	4.2 (0.1, 8.6)	0.5 (–4.4, 5.6)	2.8 (–1.3, 7.1)	1.9 (–2.6, 6.6)	1.4 (–2.2, 5.1)	3.5 (–1.5, 8.8)
Progressive motility, %c	–0.029 (–0.851, 0.792)	–0.293 (–1.132, 0.547)	–0.03 (–0.991, 0.932)	–0.356 (–1.127, 0.414)	0.128 (–0.745, 1.002)	0.136 (–0.612, 0.884)	0.542 (–0.445, 1.529)
Total motility, %d	–0.007 (–0.097, 0.084)	–0.042 (–0.134, 0.051)	0.003 (–0.104, 0.110)	–0.018 (–0.104, 0.067)	0.010 (–0.085, 0.105)	0.009 (–0.072, 0.091)	0.050 (–0.060, 0.160)
Morphologically normal spermatozoa, %	1.2 (–0.7, 3.0)	2.1 (0.3, 4.0)^*^	–0.2 (–2.5, 2.0)	1.6 (–0.2, 3.5)	1.6 (–0.2, 3.5)	0.5 (–1.3, 2.3)	1.4 (–0.9, 3.7)
Reproductive hormones							
E2 (pg/ml)	–6.5 (–9.8, –3.0)^**^	–6.7 (–10.4, –2.8)^**^	1.2 (–3.3, 5.8)	–2.1 (–5.5, 1.6)	–1.6 (–5.5, 2.5)	–1.8 (–5.3, 1.8)	0.5 (–4.0, 5.1)
FSH (mIU/ml)	2.6 (0.2, 4.7)^*^	1.9 (–0.5, 4.2)	0.5 (–2.3, 3.3)	1.4 (–0.7, 3.5)	2.1 (–0.2, 4.7)	0.0 (–1.8, 2.1)	0.2 (–2.5, 3.0)
LH(mIU/ml)	0.5 (–1.4, 2.6)	–1.1 (–3.2, 0.9)	–0.5 (–2.7, 1.9)	–0.5 (–2.3, 1.4)	0.0 (–2.1, 2.1)	0.9 (–0.9, 2.6)	–0.9 (–3.4, 1.4)
PRL (ng/ml)	0.5 (–1.6, 2.8)	–1.4 (–3.6, 0.9)	–0.2 (–2.9, 2.6)	–1.4 (–3.4, 0.7)	–1.1 (–3.4, 1.2)	–0.7 (–2.7, 1.4)	0.0 (–2.9, 2.8)
P (ng/ml)	0.2 (–3.6, 4.2)	–2.3 (–5.8, 1.6)	–0.9 (–5.2, 3.5)	0.7 (–2.9, 4.2)	0.9 (–3.2, 5.0)	–1.6 (–4.9, 1.6)	0.7 (–3.8, 5.4)
T (ng/ml)	–0.5 (–1.8, 0.9)	0.2 (–1.4, 1.6)	1.4 (–0.5, 3.3)	0.7 (–0.7, 2.1)	0.5 (–1.1, 2.1)	0.0 (–1.4, 1.4)	0.2 (–1.6, 2.3)

^a^Because semen parameters (except progressive motility and total motility) and reproductive hormones were in right-skewed distribution, they were analyzed on a logarithmic scale and back-transformed to obtain the percent change (with 95% Confidence Interval (CI) given in the brackets) for each parameter.

^b^Adjusted for age, abstinence time, smoke, alcohol and intake of tea, coffee and cola.

^c^Beta coefficient (95% CI) for progressive motility.

^d^Total motility was classified into four groups based on its quartiles. We performed multiple ordinal logistic regression analysis. Estimates and 95% CI were given.

^*^P < 0.05, ^**^P < 0.01.

Among the six reproductive hormones, we found E_2_ and FSH to be associated with pubertal timing. An one-year increase in the onset age of height spurt and peak height velocity was associated with a 6.5% and 6.7% decrease in E_2_ (95% CI = –9.8 to –3.0, –10.4 to –2.8; respectively). Further, an one-year increase in onset age of height spurt was associated with higher FSH (% change = 2.6, 95% CI = 0.2 to 4.7). The other pubertal events were found to be unrelated to reproductive hormones.

We set focus on the onset ages of pubertal events, including height spurt, peak height velocity, and first spermatorrhea. Considering Body Mass Index (BMI) and testicular volume as potential mediators, we adjusted for BMI grade and testicular volume separately in further analyses (Table 4, Models I and II). We found that sperm concentration was positively associated with the onset ages of peak height velocity and first spermatorrhea, and it persisted in all three models. The negative association between onset age of first spermatorrhea and semen volume were consistent across three models. E_2_ was negatively associated with the onset ages of height spurt and peak height velocity across all three models. Further, the positive association between onset age of height spurt and FSH was marginally significant (*P* = 0.052), after adjusting for testicular volume.

**Table 4:** Further analyses on the associations between onset ages of pubertal events, semen quality and reproductive hormones

Characteristics^a, b^	Height spurt, % Change (95% CI)		Peak height velocity, % Change (95% CI)		First spermatorrhea, % Change (95% CI)	
	model I^c^	model II^c^	model I^c^	model II^c^	model I^c^	model II^c^
Semem quality						
Sperm concentration, millions/ml	3.3 (–0.4, 7.1)	2.8 (–0.8, 6.6)	4.7 (1.0, 8.6)^*^	4.7 (0.5, 9.1)^*^	6.4 (1.7, 11.3)^**^	7.2 (2.4, 12.1)^**^
Semen volume, ml	–0.2 (–2.5, 2.0)	–0.5 (–2.7, 1.8)	0.0 (–2.2, 2.3)	0.2 (–2.0, 2.5)	–2.9 (–5.5, –0.3)^*^	–3.6 (–6.2, –1.0)^*^
Total sperm count, millions	2.3 (–1.7, 6.6)	1.6 (–2.4, 5.8)	4.2 (0.1, 8.6)	4.2 (0.1, 8.6)	3.5 (–1.5, 8.8)	3.5 (–1.9, 9.3)
Progressive motility, %	–0.046 (–0.868, 0.777)	–0.222 (–1.056, 0.613)	–0.308 (–1.149, 0.533)	–0.339 (–1.189, 0.511)	0.542 (–0.445, 1.529)	0.6 (–0.434, 1.633)
Total motility, %	0.0 (–0.5, 0.5)	0.0 (–0.5, 0.5)	0.0 (–0.5, 0.4)	0.0 (–0.9, 0.9)	0.5 (–0.4, 1.4)	0.7 (–0.2, 1.6)
Morphologically normal spermatozoa, %	0.7 (–1.1, 2.5)	1.2 (–0.7, 3.0)	1.4 (–0.4, 3.2)	1.9 (0.0, 3.7)	1.4 (–0.9, 3.7)	1.2 (–1.1, 3.5)
Reproductive hormones						
E_2_ (pg/ml)	–6.5 (–9.8, –2.9)^**^	–7.3 (–10.7, –3.6)^**^	–6.7 (–10.1, –2.9)^**^	–7.7 (–11.3, –4.1)^**^	0.5 (–4.1, 5.0)	–1.8 (–6.2, 2.8)
FSH (mIU/ml)	2.6 (0.2, 5.0)^*^	2.3 (0.0, 4.5)	1.9 (–0.5, 4.2)	1.6 (–0.7, 4.0)	0.2 (–2.5, 3.0)	0.2 (–2.5, 3.3)
LH (mIU/ml)	0.7 (–1.4, 2.6)	0.2 (–1.6, 2.3)	–0.7 (–2.7, 1.4)	–1.4 (–3.4, 0.7)	–0.9 (–3.4, 1.4)	–1.4 (–4.1, 1.2)
PRL (ng/ml)	0.5 (–1.6, 2.8)	–0.2 (–2.5, 2.1)	–1.1 (–3.6, 1.2)	–1.6 (–4.1, 0.7)	0.0 (–2.9, 2.8)	–0.7 (–3.6, 2.3)
P (ng/ml)	0.2 (–3.6, 4.2)	–0.2 (–4.1, 4.0)	–2.9 (–6.7, 0.9)	–2.7 (–6.7, 1.2)	0.5 (–4.1, 5.2)	1.2 (–3.8, 6.4)
T (ng/ml)	–0.7 (–2.3, 0.7)	–0.7 (–2.3, 0.9)	–0.2 (–1.8, 1.2)	0.0 (–1.4, 1.6)	0.2 (–1.6, 2.1)	0.2 (–1.8, 2.1)

^a^Because semen parameters (except progressive motility) and reproductive hormones were in skewed distribution, they were analyzed on a logarithmic scale and back-transformed to obtain the percent change (with 95% Confidence Interval (CI) given in the brackets) for each parameter. Beta coefficient (95% CI) for progressive motility.

^b^Adjusted for age, abstinence time, smoke, alcohol and intake of tea, coffee and cola.

^c^Model I: additionally adjusted for BMI grade. Model II: additionally adjusted for testicular volume.

^*^P < 0.05, ^**^P < 0.01.

Pubertal timing variables were classified into four groups by quartiles. Setting Q1 as the reference, subjects in the Q4 group had higher sperm concentration (20.8% (95% CI = 2.1 to 42.9) and 29.7% (95% CI = 5.2 to 60.0) for onset age of peak height velocity and first spermatorrhea, respectively) (Table 5). Subjects in the Q4 group had lower E_2_ (**−**22.4% (95% CI = –34.2 to –8.4) and –22.4% (95% CI= –34.7 to –7.7) for onset age of height spurt and peak height velocity, respectively) (Table 5).

**Table 5:** Associations between onset ages of pubertal events, semen quality and reproductive hormones (categorical variables)

Onset ages of pubertal events^a^		*N*	Sperm concentration^b,c^, % change (95% CI)	Semen volume^b,c^, % change (95% CI)	Morphologically normal spermatozoa^b,c^, % change (95% CI)	E2^b,c^, % change (95% CI)	FSH^b,c^, % change (95% CI)
Height spurt	Q1	275	Reference	Reference	Reference	Reference	Reference
	Q2	101	11.7 (–6.0, 32.7)	3.8 (–6.2, 14.8)	6.9 (–2.1, 16.7)	–6.9 (–21.7, 10.9)	–0.5 (–10.5, 10.4)
	Q3	120	3.5 (–11.9, 21.9)	2.3 (–7.1, 12.5)	4.7 (–3.6, 13.5)	–10.7 (–23.8, 5.0)	4.2 (–5.4, 14.8)
	Q4	114	10.2 (–6.7, 30.0)	–2.1 (–11.3, 7.9)	–0.9 (–8.8, 7.6)	–22.4 (–34.2, –8.4)^**^	14.0 (3.3, 25.9)^**^
Peak height velocity	Q1	193	Reference	Reference	Reference	Reference	Reference
	Q2	154	15.1 (–1.8, 34.9)	6.2 (–3.4, 16.4)	10.9 (2.1, 20.2)^*^	–5.8 (–20.0, 10.9)	–2.3 (–11.3, 7.6)
	Q3	137	13.5 (–3.8, 33.7)	–4.7 (–13.5, 4.7)	5.2 (–3.2, 14.3)	–17.0 (–29.9, –1.6)^*^	–0.2 (–9.6, 10.4)
	Q4	127	20.8 (2.1, 42.9)^*^	3.3 (–6.5, 14.0)	6.4 (–2.3, 15.6)	–22.4 (–34.7, –7.7)^**^	7.9 (–2.7, 19.7)
First spermatorrhea	Q1	198	Reference	Reference	Reference	Reference	Reference
	Q2	143	3.8 (–11.7, 22.2)	0.5 (–8.6, 10.4)	–1.4 (–9.2, 7.2)	–3.8 (–17.8, 12.2)	4.0 (–5.8, 14.8)
	Q3	91	4.5 (–13.3, 25.9)	–7.7 (–17.2, 3.0)	0.0 (–9.0, 9.9)	–1.1 (–17.6, 18.6)	–1.6 (–12.3, 10.2)
	Q4	65	29.7 (5.2, 60.0)^*^	–8.8 (–19.5, 3.3)	4.7 (–5.8, 16.4)	0.7 (–18.2, 24.2)	8.6 (–4.7, 23.6)

^a^Pubertal timing variables were classified into quartiles (Q1-Q4, from the lowest group to the highest group).

^b^Because semen parameters and reproductive hormones were in right-skewed distribution, they were analyzed on a logarithmic scale and back-transformed to obtain the percent change (with 95% confidence interval given in the brackets) for each parameter.

^c^Adjusted for age, abstinence time, smoke, alcohol and intake of tea, coffee and cola.

^*^P < 0.05, ^**^P < 0.01.

## DISCUSSION

To the best of our knowledge, this was the first large-scale designed study to explore the associations between pubertal timing, semen quality and reproductive hormones. In this population of 680 male college students, we found that early pubertal timing was associated with lower sperm concentration. Pubertal timing was also associated with alterations in E_2_ and FSH. These results were independent of several potential confounders that included age, abstinence time, smoke, etc.

There are several explanations for these observed associations between pubertal timing and semen parameters. First, some studies have shown an association between early pubertal timing and higher BMI in adulthood [4]. The negative association between semen quality and BMI has been well characterized [22, 23], total sperm number and sperm concentration were the two parameters found to be more strongly related to obesity [24]. We then hypothesized whether the observed associations between pubertal timing and semen parameters were mediated by BMI. To verify this hypothesis, we additionally adjusted for BMI in the regression analyses. The results were consistent. Besides, the median of BMI in our study was 20.9 kg/m^2^ (percentile 25th to percentile 75th = 19.6 to 22.7), and only 2.6% of the subjects were defined as obese, which is to say this conclusion was drawn from a population of lean men. The possibility of BMI as a mediator between pubertal timing and semen quality, therefore, still exists. Further studies were needed to demonstrate the role of BMI. Secondly, our findings may reflect the physiologic effect of pubertal timing. Puberty is an important transformation period, as one moves from childhood to adulthood and reproductive maturity. During this period, the HPG axis was triggered for sexual maturation leading to culminations in adult hormonal profiles and physical changes like secondary sexual characteristics [2]. In terms of testis development, the growth of testicular volume during puberty is a marker for spermatogenesis. Stimulation of leydig cells by LH will lead to an increase of testosterone. The level of testosterone will influence the growth of penis in terms of its width and length. The most important change in the end is the achievement of fertility. One possible mechanism is that pubertal timing is an indicative marker for the neuroendocrine system, which regulates the development of reproductive system. Lastly, pubertal timing in this study was negatively associated with E_2_ and positively associated with FSH. E_2_ plays a pivotal role in male reproductive health. It has been confirmed that both testicular somatic cells and germ cells are sources of estrogen in mammals including men [17]. So the level of E_2_ does reflect the general functionality of these two cell types. Exposure of the testis to extra estrogen contributes to the decline of sperm concentration [25, 26]. Pubertal timing can thus be regarded as an indicative marker for hormone levels in men in the future.

Although some important discoveries were revealed in this study, there were also certain limitations. First, only a single semen sample was obtained from each volunteer. Using only a single semen sample to predict male reproductive health over a long period is not accurate, even though collecting only one semen sample is acceptable in population-based epidemiological studies. Another limitation was the data collecting. Studies on the pubertal timing of girls are much more than that of boys. In common practice, menarche is considered as a gold standard for the measurement of pubertal timing in girls. However, it is still debated how to measure pubertal development more accurately in boys and how to incorporate it among different researches.

To deal with this methodological shortage, we used several methods to reduce the retrospective bias as follows: 1. The questionnaire was modified from those used in others’ studies. The Pubertal Development Scale (PDS) uses self-reports on volunteers’ development [27, 28]. It is a practical questionnaire to use in retrospective epidemiological studies. In addition, Felix R. Day [3] and Anders Juul [29] used the age of voice breaking as a single marker of pubertal timing. Additionally, the age of first spermarche was also regarded as a convenient marker in population-based studies [30]. We integrated these items to form our modified questionnaire. 2. High education background and young age were sufficient enough for the volunteers to give answers close to the reality. The options of “not clear” and “reject to answer” were both alternatives for the participants. 3. Volunteers were not informed about their test results before responding to the questionnaire, so it would not affect their responses. 4. We created a grade-age look-up table to help the volunteers answer their corresponding age more precisely. In a pilot investigation, we found that some volunteers were more sensitive to their grades than ages when recalling a specific pubertal event. The grade-age look-up table was listed on the same page with the questionnaire, as that made it convenient for volunteers to use this table as a reference.

The questionnaire for the data collection was validated by several methods. First, we compared the data on pubertal timing to those of healthy Chinese children. Fang *et al*. [31] reported that the mean onset ages of body hair growth and first spermatorrhea were 13.6 years in Chongqing, China. Similarly, our results were 13.5 years and 13.8 years, respectively. In a cross-sectional study of 18, 807 urban Chinese boys [32], the median onset ages of body hair growth and first spermatorrhea were 12.8 years and 14.0 years. Similarly, our results were 13.0 years and 14.0 years. In addition, the age of first spermatorrhea in our study was consistent with the result of Chinese National Surveys on Students Constitution and Health (14.0 years). Second, we analyzed the inter-correlations between these pubertal timing markers. The Spearman correlation coefficients ranged from 0.356 to 0.738, indicating that the onset ages of pubertal events did significantly correlate with each other. Third, the overall raw Cronbach's alpha coefficient was 0.886, indicating that the data on pubertal timing was of high consistency.

The article by Jensen *et al*. [33] provided an opportunity for comparison between different studies. Theirs was a cross-sectional study that investigated the association between pubertal timing and subsequent reproductive health among 1068 young Danish men. The study reported that later onset of puberty was associated with declined semen quality, a finding different from our results. While we found similarities for reproductive hormones. FSH was positively associated with pubertal timing. The difference between these two studies may have been due to the different methods used for data collecting. The subjects of their study were divided into three groups, defined as earlier, the same as, and later than peers. On the other hand, the differences between the studies indicated a necessity to validate the association between pubertal timing and reproductive health. Prospective study was a better way to solve the methodological shortage in the present study.

In conclusion, for the first time, we found that early pubertal timing was associated with lower semen concentration. In addition, pubertal timing was associated with alterations in reproductive hormones. The result should be interpreted with cautious. Pubertal timing may be a potential marker of semen quality in adulthood. Larger and more diverse populations are still needed to draw and confirm a solid conclusion.

## MATERIALS AND METHODS

### Study population

This survey was conducted in June of 2013. Young male volunteers were recruited from colleges in Chongqing China. Individuals were excluded if they met any of the following criteria: <18 years old; <2 or >7 days of abstinence time; a history of inflammation of the urogenital system, epididymitis or testicular injury; a history of undescended testis; or a history of varicocele treatment; or if any of the following were detected by an urologist at the physical examination stage of the investigation: an absence of prominentia laryngea, pubis or testis; abnormal breasts or penis; epididymal knob; or varicocele. Both semen and reproductive hormones were analyzed for the remaining participants. The participants completed a questionnaire and also underwent a physical examination. All detailed data regarding study design, data acquisition, etc., were published previously [18].

According to the above criteria, 796 volunteers were eligible for this survey, and 116 volunteers were excluded because of their refusal to answer questions on puberty. In the end, therefore, a total of 680 volunteers were include in our study.

This study was approved by the Ethics Committees of Third Military Medical University. The experiment methods were carried out in accordance with the approved guidelines. Informed consent was also obtained from each subject.

### Questionnaire

Upon entry, all subjects completed a questionnaire consisting of medical history, lifestyle, mental stress, and so on. Information on potential confounders including age, abstinence time, BMI, smoke, alcohol, intake of tea, Coke and coffee was also collected on the questionnaire. To improve the reliability and validity of the data on pubertal timing, we developed a questionnaire based on PDS [28, 34] and Sexual Maturation Scale (SMS) [35]. The participants were asked to identify pubertal events and then answer the age of first onset. The questions included in our questionnaire were the following: 1. Was your own pubertal timing early, average, or late compared to your peers? 2. How old were you when your height started to spurt? 3. How old were you when your growth velocity was the highest? 4. How old were you when you noticed that your body hair started to grow? 5. How old were you when you noticed changes in your skin (such as acne, oily skin, rough pore and rough skin)? 6. How old were you when you noticed your voice deepening? 7. How old were you when you noticed your facial hair started to grow? 8. What was the age of your first spermatorrhea? A grade-age look-up table (Supplementary Table 1) was supplied to help volunteers to answer the ages of these pubertal related events more precisely.

### Physical examination

Height and weight were measured, and BMI was calculated as weight in kilograms divided by height squared in meters. Additionally, an experienced urologist will test the presence of varicocele and measure testicular volume by Prader's orchidometer (FUAN enterprise, Shanghai, China).

### Semen collection and analysis

The volunteers had been instructed to keep abstinence time between 2 to 7 days, and their abstinence time was recorded on the questionnaire. Semen samples were collected by masturbation into a wide-mouth plastic container at a private room. Then it was incubated at 37°C for liquefaction. An experienced technician performed semen analysis in accordance with the World Health Organization 2010 guideline [15]. Semen volume was assessed by weighing semen sample assuming 1g = 1ml. Sperm morphology was identified by sperm smears using Diff-Quick staining (Bred life science, Product code: BRED-015). Sperm concentration and motility were assessed by computer-aided sperm analysis (SCA CASA System; Microptic S.L., Barcelona, Spain). Sperm motility was classified as progressive motility and total motility.

### Hormone analysis

Blood sample was withdrawn from an antecubital vein. Serum was separated and frozen at –80°C until assay. Reproductive hormones including testosterone (T), estrogen (E_2_), progesterone (P), follicle-stimulating hormone (FSH), luteinizing hormone (LH), and prolactin (PRL) were determined at the clinical laboratory of Southwest Hospital (Chongqing, China) by Beckman Unicel DXI 800 Immunoassay System (Beckman Coulter Inc., California, US). The detection range was 1.2–187.1 mIU/mL, 0.7–85.9 mIU/mL, 0.4–30.4 ng/mL, 0.6–75.7 ng/mL, 20–4233 pg/mL and 0.1–14.8 ng/mL for FSH, LH, P, PRL, E_2_ and T, respectively. The intra-assay coefficient of variation was 3.5%, 3.8%, 6.1%, 1.6%, 12% and 3.9% for FSH, LH, P, PRL, E_2_ and T, respectively.

### Statistical analysis

Volunteers were classified according to the percentile of onset age of peak height velocity. There were four groups: total, ≤25th percentile, 25th-75th percentile and >75th percentile. The Kruskal-Wallis tests and Chi-square tests were used to evaluate differences in basic characteristics between the groups (kruskal-Wallis tests for continuous variables and Chi-square tests for categorical variables). Parameters were presented as median (25th to 75th percentile) or numbers (percentages). Several descriptive variables (mean, standard deviation, median, percentile 25 and percentile 75) were calculated for the pubertal variables. The inter-correlations between these variables were analyzed using the Spearman correlation. Cronbach's alpha was used to quantify the internal consistency of the pubertal variables in this survey.

Most of the data on semen parameters and reproductive hormones were right-skewed (except progressive motility in normal distribution). We transformed these data into a logarithmic scale before analysis. We used multivariate linear regression models to explore the associations between pubertal timing, semen quality and reproductive hormones. To facilitate the interpretation of the data, the results were back transformed and quantified as a percent change (95% confidence interval (CI)). Percent change = (10^β^-1)*100%. Total motility (left-skewed) was classified into four groups based on its quartiles. We performed multiple ordinal logistic regression analysis. Total motility were recoded into 1 to 4 from Q1 to Q4.

The factors that were possibly associated with semen quality and pubertal timing were selected as potential confounders: Age, abstinence time, smoke (never, ever, current), alcohol (never, ever, current), intake of tea (never, ever, current), Coke (0 cups/week, <3 cups/week, 3–6 cups/week, >6 cups/week) and coffee (0 cups/week, <3 cups/week, 3–6 cups/week, >6 cups/week). Tea intake was categorized differently than Coke and coffee, because the percentage of people who chose “ever” and “current” for tea intake were similar (16.5% and 19.0%, respectively). These were adjusted for in the regression model by stepwise method. In addition, BMI in adulthood was reported to be associated with pubertal timing [4] and semen quality [22, 23]. In biology, BMI may be in the causal pathway between pubertal timing and reproductive outcomes. As a result, BMI (< 18.5 kg/m^2^, 18.5–23.9 kg/m^2^, 24–27.9 kg/m^2^, ≥28 kg/m^2^) was regarded as a potential mediator and adjusted for along with other potential confounders. Adult testicular volume is achieved at the end of puberty. It occurs temporally after the onset of puberty and is a well-known predictor for reproductive outcomes [36]. Thus, it was considered as a mediator and adjusted for along with other potential confounders. It should be noted as well that season and temperature were not adjusted for, because this survey was conducted during a short period in the summer. In addition, three pubertal timing variables were classified into quartiles based on its distribution in the subjects. Regression coefficients of Q2, Q3 and Q4 were calculated, with Q1 as the reference level.

The *P* value <0.05 was considered significant. All the statistical analyses were performed using SPSS 18.0 (IBM).

## SUPPLEMENTARY MATERIALS TABLES



## Abbreviations

HPG: hypothalamic-pituitary-gonadal
MARHCS: Male Reproductive Health in Chongqing College Students
PDS: Pubertal Development Scale
SMS: Sexual Maturation Scale
BMI: body mass index
T: testosterone
E_2_: estrogen
P: progesterone
FSH: follicle-stimulating hormone
LH: luteinizing hormone
PRL: prolactin
CNSSCH: Chinese National Surveys on Students Constitution and Health
CI: 95% confidence interval

## References

[1] Cousminer DL, Stergiakouli E, Berry DJ, Ang W, Groen-Blokhuis MM, Korner A, Siitonen N, Ntalla I, Marinelli M, Perry JR, Kettunen J, Jansen R, Surakka I (2014). Genome-wide association study of sexual maturation in males and females highlights a role for body mass and menarche loci in male puberty. Hum Mol Genet.

[2] Zawatski W, Lee MM (2013). Male pubertal development: are endocrine-disrupting compounds shifting the norms?. J Endocrinol.

[3] Day FR, Elks CE, Murray A, Ong KK, Perry JR (2015). Puberty timing associated with diabetes, cardiovascular disease and also diverse health outcomes in men and women: the UK Biobank study. Sci Rep.

[4] Prentice P, Viner RM (2013). Pubertal timing and adult obesity and cardiometabolic risk in women and men: a systematic review and meta-analysis. Int J Obes.

[5] Widen E, Silventoinen K, Sovio U, Ripatti S, Cousminer DL, Hartikainen AL, Laitinen J, Pouta A, Kaprio J, Jarvelin MR, Peltonen L, Palotie A (2012). Pubertal timing and growth influences cardiometabolic risk factors in adult males and females. Diabetes Care.

[6] Golub MS, Collman GW, Foster PM, Kimmel CA, Rajpert-De Meyts E, Reiter EO, Sharpe RM, Skakkebaek NE, Toppari J (2008). Public health implications of altered puberty timing. Pediatrics.

[7] Biro FM, Deardorff J (2013). Identifying opportunities for cancer prevention during preadolescence and adolescence: puberty as a window of susceptibility. J Adolesc Health.

[8] Mantovani A, Fucic A (2014). Puberty dysregulation and increased risk of disease in adult life: possible modes of action. Reprod Toxicol.

[9] Richiardi L, Askling J, Granath F, Akre O (2003). Body size at birth and adulthood and the risk for germ-cell testicular cancer. Cancer Epidemiol Biomarkers Prev.

[10] Zhou X, Zhang L (2015). The Trends of Puberty Onset among Chinese Children. Iran J Public Health.

[11] Jaruratanasirikul S, Sriplung H (2015). Secular trends of growth and pubertal maturation of school children in Southern Thailand. Ann Hum Biol.

[12] Carlsen E, Giwercman A, Keiding N, Skakkebaek NE (1992). Evidence for decreasing quality of semen during past 50 years. BMJ.

[13] Swan SH, Elkin EP, Fenster L (1997). Have sperm densities declined? A reanalysis of global trend data. Environ Health Perspect.

[14] Bonde JP, Ernst E, Jensen TK, Hjollund NH, Kolstad H, Scheike T, Giwercman A, Skakkebæk NE, Henriksen TB, Olsen J (1998). Relation between semen quality and fertility: a population-based study of 430 first-pregnancy planners. Lancet (London, England).

[15] WHO (2010). WHO Laboratory Manual for the Examination and Processing of Human Semen.

[16] Dohle GR, Smit M, Weber RF (2003). Androgens and male fertility. World J Urol.

[17] Carreau S, Lambard S, Delalande C, Denis-Galeraud I, Bilinska B, Bourguiba S (2003). Aromatase expression and role of estrogens in male gonad. Rev Reprod Bio and Endo.

[18] Yang H, Chen Q, Zhou N, Sun L, Bao H, Tan L, Chen H, Zhang G, Ling X, Huang L, Li L, Ma M, Yang H (2015). Lifestyles Associated With Human Semen Quality: Results From MARHCS Cohort Study in Chongqing, China. Medicine.

[19] Chen Q, Yang H, Zhou N, Sun L, Bao H, Tan L, Chen H, Ling X, Zhang G, Huang L, Li L, Ma M, Yang H (2016). Inverse U-shaped Association between Sleep Duration and Semen Quality: Longitudinal Observational Study (MARHCS) in Chongqing, China. Sleep.

[20] Zhou N, Sun L, Yang H, Chen Q, Wang X, Yang H, Tan L, Chen H, Zhang G, Ling X, Huang L, Zou P, Peng K (2016). Anogenital distance is associated with serum reproductive hormones, but not with semen quality in young men. Hum Reprod.

[21] Zhang G, Yan H, Chen Q, Liu K, Ling X, Sun L, Zhou N, Wang Z, Zou P, Wang X, Tan L, Cui Z, Zhou Z (2016). Effects of cell phone use on semen parameters: Results from the MARHCS cohort study in Chongqing, China. Environ Int.

[22] Cabler S, Agarwal A, Flint M, du Plessis SS (2010). Obesity: modern man's fertility nemesis. Asian J Androl.

[23] Ehala-Aleksejev K, Punab M (2015). The different surrogate measures of adiposity in relation to semen quality and serum reproductive hormone levels among Estonian fertile men. Andrology.

[24] Sermondade N, Faure C, Fezeu L, Shayeb AG, Bonde JP, Jensen TK, Van Wely M, Cao J, Martini AC, Eskandar M, Chavarro JE, Koloszar S, Twigt JM (2013). BMI in relation to sperm count: an updated systematic review and collaborative meta-analysis. Hum Reprod Update.

[25] Handelsman DJ (2001). Estrogens and falling sperm counts. Reprod Fertil Dev.

[26] Lombardi G, Zarrilli S, Colao A, Paesano L, Di Somma C, Rossi F, De Rosa M (2001). Estrogens and health in males. Mol Cell Endocrinol.

[27] Bond L, Clements J, Bertalli N, Evans-Whipp T, McMorris BJ, Patton GC, Toumbourou JW, Catalano RF (2006). A comparison of self-reported puberty using the Pubertal Development Scale and the Sexual Maturation Scale in a school-based epidemiologic survey. J Adolesc.

[28] Yousefi M, Karmaus W, Zhang H, Roberts G, Matthews S, Clayton B, Arshad SH (2013). Relationships between age of puberty onset and height at age 18 years in girls and boys. World J Pediatr.

[29] Juul A, Magnusdottir S, Scheike T, Prytz S, Skakkebaek NE (2007). Age at voice break in Danish boys: effects of pre-pubertal body mass index and secular trend. Int J Androl.

[30] Song Y, Ma J, Li LB, Dong B, Wang Z, Agardh A (2016). Secular trends for age at spermarche among Chinese boys from 11 ethnic minorities, 1995-2010: a multiple cross-sectional study. BMJ Open.

[31] Fang Q, Wang H, Cao X, Chen H (2012). Puberty timing status and its correlation with childhood obesity in Chongqing City [Article in Chinese]. Wei Sheng Yen Chiu.

[32] Ma HM, Chen SK, Chen RM, Zhu C, Xiong F, Li T, Wang W, Liu GL, Luo XP, Liu L, Du ML, Pubertal Study Group of the Society of Pediatric Endocrinology and Genetic Disease, Chinese Medical Association (2011). Pubertal development timing in urban Chinese boys. Int J Androl.

[33] Jensen TK, Finne KF, Skakkebaek NE, Andersson AM, Olesen IA, Joensen UN, Bang AK, Nordkap L, Priskorn L, Krause M, Jorgensen N, Juul A (2016). Self-reported onset of puberty and subsequent semen quality and reproductive hormones in healthy young men. Hum Reprod.

[34] Petersen AC, Crockett L, Richards M, Boxer A (1988). A self-report measure of pubertal status: Reliability, validity, and initial norms. J Youth Adolesc.

[35] Marshall WA, Tanner JM (1970). Variations in the pattern of pubertal changes in boys. Arch Dis Child.

[36] Hart RJ, Doherty DA, McLachlan RI, Walls ML, Keelan JA, Dickinson JE, Skakkebaek NE, Norman RJ, Handelsman DJ (2015). Testicular function in a birth cohort of young men. Hum Reprod.

