# Supplementary material for: Early pubertal timing is associated with lower sperm concentration in college students
Source: Oncotarget. 2018 Feb 6;9(36):24178–86. doi: 10.18632/oncotarget.24415 (PMC5966273; doi:10.18632/oncotarget.24415)
Supplement: Supplementary file 1 [file oncotarget-09-24178-s001.pdf]

Early pubertal timing is associated with lower sperm concentration in college students

SUPPLEMENTARY MATERIALS

Supplementary Table 1: Look-up table for grade and age

Birthday	Grade-6	Grade-7	Grade-8	Grade-9	High school-1	High school-2	High school-3	College-1	College-2	College-3
1996.6.1-	9	10	11	12	13	14	15	16	17	18
1995.6.1-	10	11	12	13	14	15	16	17	18	19
1994.6.1-	11	12	13	14	15	16	17	18	19	20
1993.6.1-	12	13	14	15	16	17	18	19	20	21
1992.6.1-	13	14	15	16	17	18	19	20	21	22

The grade-age look-up table was used to help volunteers recall corresponding ages more precisely. For example, a boy recalled that his height started to spurt when he was in grade 7, and he was born between 1993-6-1 and 1994-6-1. Then his age of height spurt was 13 years old.

Supplementary Table 2: Spearman correlations of onset ages of pubertal events

Onset age of pubertal events (year)		Peak height velocity	Body hair growth	Skin changes	Voice deepening	Facial hair growth	First spermatorrhea
Height spurt	Coefficient	0.691**	0.480**	0.507**	0.450**	0.356**	0.465**
	P	<0.001	<0.001	<0.001	<0.001	<0.001	<0.001
	N	587	508	515	507	519	466
Peak height velocity	Coefficient		0.567**	0.558**	0.586**	0.437**	0.451**
	P		<0.001	<0.001	<0.001	<0.001	<0.001
	N		513	518	514	522	468
Body hair growth	Coefficient			0.613**	0.623**	0.554**	0.591**
	P			<0.001	<0.001	<0.001	<0.001
	N			495	486	509	456
Skin changes	Coefficient				0.738**	0.603**	0.523**
	P				<0.001	<0.001	<0.001
	N				498	511	458
Voice deepening	Coefficient					0.633**	0.542**
	P					<0.001	<0.001
	N					512	447
Facial hair growth	Coefficient						0.458**
	P						<0.001
	N						467

**Correlation is significant at the 0.01 level (2-tailed).
